# Effect of Different Fineness of Cement on the Autogenous Shrinkage of Mass Concrete under Variable Temperature Conditions

**DOI:** 10.3390/ma16062367

**Published:** 2023-03-15

**Authors:** Jiale Gong, Zhongyang Mao, Zhe Cao, Xiaojun Huang, Min Deng

**Affiliations:** 1 College of Materials Science and Engineering, Nanjing Tech University, Nanjing 211800, China; 2 State Key Laboratory of Materials-Oriented Chemical Engineering, Nanjing 211800, China

**Keywords:** shrinkage of concrete, total volume deformation, different temperature stage, cement paste

## Abstract

The internal temperature of the mass concrete is not constant. In the actual project, the internal temperature of the concrete will experience a process of rapid warming to reach the peak temperature and then slow down. In this study, volume measurement method is used to simulate the internal volume deformation of mass concrete under actual engineering conditions. The embedded strain gauge was embedded in concrete with a water–cement ratio of 0.32 for 28 days, and the development of total volume deformation and autogenous volume deformation of concrete under variable temperature conditions was studied by external heating of concrete. The results show that the finer the cement, the earlier the concrete starts to shrink, and the greater the total shrinkage of the concrete. The high temperature will promote the hydration of cement with different fineness and will reduce the total porosity of their paste, but the proportion of harmful pores in the paste with finer cement particles is lower.

## 1. Introduction

With the rapid development of the economy and technology, mass concrete is more and more widely used in modernizing industrial societies because of its outstanding structural strength and durability. In the world, many high-rise buildings, railways, highways, bridges, urban road, subways, dam power stations, airports, shopping malls, and other infrastructure projects that require mass concrete have emerged [1,2,3].

There are currently no uniform quantitative regulations for mass concrete in scientific institutions around the world. American Concrete Institute (ACI) regulations: regardless of the concrete poured in any site, due to the size of the concrete volume being too large, the heat of hydration generated by the concrete must be strictly controlled to reduce the volume of deformation problems due to heat of hydration to minimize the concrete cracking situation, which is called mass concrete. The Ministry of Housing and Urban-Rural Development of China stipulates that two rules can classify mass concrete. One is bulk concrete with a minimum geometry of concrete structural entities of not less than 1 m. The second is expected to produce harmful cracks due to temperature changes and shrinkage caused by the hydration of the cementitious materials in the concrete. The Japanese Institute of Architects (JIA) defines mass concrete as concrete whose structural length in the section is longer than 800 mm and where the exothermic hydration of the cement results in a temperature difference between the inside and outside of the concrete of more than 25 °C. Although there is no quantitative uniformity in academia regarding mass concrete, there is some common understanding of the characteristics of mass concrete. For example, the total volume of concrete poured is large, the internal heat of hydration does not easily escape, leading to increased internal temperatures, the total shrinkage is considerable, and early cracks are likely to occur.

Cracks in concrete have different causes when concrete is at different ages. At later ages, external forces are exerted on the concrete structure in excess of what it can carry, while concrete cracks at earlier ages mainly because of problems with the concrete itself. The early cracking of concrete is because mass concrete will use a low water–cement ratio to ensure its workability. A low water–cement ratio will allow for more cement to participate in the hydration reaction, causing more significant total shrinkage and ultimately causing concrete cracking [4,5,6,7,8]. The involvement of more cement in the hydration reaction also means more heat is generated inside the concrete. Because the temperature environment inside the mass concrete is similar to adiabatic conditions [9], the lower thermal conductivity of concrete [10] prevents heat from dissipating inside mass concrete promptly, and the high temperature inside the concrete promotes the hydration reaction of cement particles [11,12,13].

Thus, both the cement’s hydration reaction and the concrete’s temperature affect the concrete’s volume shrinkage deformation. Volume shrinkage can be filled by increasing the concrete before the initial setting. As hydration continues and a strength structure is formed in the concrete, volume shrinkage will be inhibited, and stresses will be generated. The tensile stresses generated internally will then increase with the increase in modulus of elasticity. When the tensile stresses exceed the tensile strength of the concrete, penetration cracks will be generated within the concrete [14,15,16,17,18,19,20]. The occurrence of harmful cracks in mass concrete at an early stage can affect the integrity, durability, water resistance, and corrosion resistance of the entire concrete building structure. Damage to concrete due to early shrinkage occurs worldwide, threatening the safety of life and property [21,22,23,24].

We have learned that the early age cracking of mass concrete is the main reason for the volume shrinkage of the concrete, which is caused mainly by the autogenous shrinkage of the concrete. As with the definition of mass concrete, the current academic definition of autogenous shrinkage is also relatively vague. Lynam [25] was probably the first researcher to discover and clearly define the autogenous shrinkage of concrete, which he defined as shrinkage not caused by heat or water evaporation. The Japanese Concrete Association [26] defines the deformation of autogenous shrinkage as the reduction in the macroscopic volume of concrete after the initial setting, due only to the hydration of the cement itself and not to other external conditions such as changes in temperature or increases or decreases in cement quantity. Jensen [27] and Li [28] considered the phenomenon of autogenous shrinkage due to self-desiccation shrinkage caused by the decrease in relative humidity inside the concrete with the hydration of cement, resulting in the unsaturated water in the capillaries. They concluded that the autogenous shrinkage of concrete is equivalent to self-desiccation shrinkage. 

However, the current view of Sun [29], who discusses the various shrinkage mechanisms within concrete separately, is more accepted by most. The degree of macroscopic autogenous shrinkage of concrete is not a single shrinkage process but is determined by a combination of chemical shrinkage, self-desiccation shrinkage, and pore structure. Chemical shrinkage is the hydration reaction of cement minerals occurring at a constant temperature and under absolute humidity (no moisture exchange with the outside world). The main mineral compositions involved in the hydration reaction are: C_3_S, C_3_A, C_2_S, C_4_AF, and their hydration reaction equations are [30].

2C_3_S + 6H→C_3_S_2_H_3_ + 3CH (1)

2C_2_S + 4H→C_3_S_2_H_3_ + CH (2)

C_3_A + 6H→C_3_AH_6_ (3)

C_4_AF + 2CH + 10H→C_3_AH_6_ + 3C_3_FH_6_ (4)

**Figure 1:**
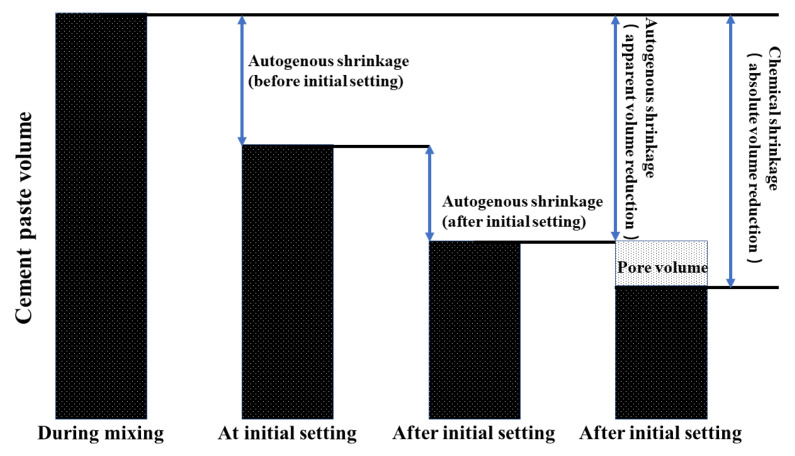
Relationship between autogenous shrinkage and chemical shrinkage.

Due to the compound’s density difference before and after the hydration reaction, the chemical shrinkage is expressed macroscopically as a reduction in absolute volume after participation in the hydration reaction. Powers quantified the chemical shrinkage of different cement clinker phases, which was 6–7 mL/(100 g) for 100% complete hydration of silicate cement and up to 20 mL/(100 g) for the incorporation of silica fume [31]. The total amount of chemical shrinkage was obtained by calculating the molecular weight and density change as the reactants react to produce the reaction products. The total volume reduction of most silicate cement pastes is 7% to 9% when fully hydrated [30]. When the cement paste in concrete is in the plastic stage, chemical shrinkage and plastic deformation together lead to a reduction in the macroscopic volume of the concrete when the water inside the concrete is free to circulate. As the concrete begins to harden after the initial and final set, the skeletal structure of the concrete gradually forms, and the water within the concrete starts to be compartmentalized, forming many water-filled pore structures. As the cement continues to hydrate and consume the water in the pores, the radius of curvature of the concave surface of the water in the pores gradually decreases, and the surface tension of the water in the capillary gradually increases, a phenomenon known as the capillary effect. The pressure generated by the capillary effect causes physical shrinkage deformation of the concrete [32], also known as self-desiccation shrinkage. However, at this point, there are still many pores inside the concrete, which disguisedly increase the volume of the concrete. Autogenous shrinkage is caused by chemical shrinkage but is not the same. The change in absolute volume is chemical shrinkage, and the reduction in appearance volume is autogenous shrinkage, so chemical shrinkage, self-desiccation shrinkage, and pore structure together determine the degree of macroscopic autogenous shrinkage of concrete, as shown in Figure 1.

However, the above definition of concrete autogenous shrinkage still has some limitations. Because the temperature generated inside the concrete does not conduct quickly in large volumes, there is a process of temperature change inside the concrete that rises rapidly and then falls slowly, and both autogenous shrinkage and thermal expansion are significantly correlated with temperature change [33,34,35,36]. Therefore, the autogenous shrinkage studied at a constant temperature does not indicate the true autogenous shrinkage within the concrete. At the same time, the fineness parameters of the cement will also influence the chemical shrinkage process and thus lead to a different degree of autogenous shrinkage in the concrete. The size of the cement particles affect the rate of hydration reaction, resulting in different rates of exothermic hydration in the early stages, and the different temperatures cause differences in the degree of autogenous shrinkage of the concrete. For the concrete autogenous shrinkage test experimental results, most are currently in the laboratory at 20 or 60–80 degrees Celsius constant temperature and humidity curing conditions, but with such constant temperature curing, the actual project has experienced a variable temperature course of the concrete internal temperature conditions. These are different and cannot be directly applied to the actual project.

In this experiment, four different fineness grades of cement were cast into concrete specimens and placed in a variable temperature curing chamber, which was externally heated to simulate the temperature change from rapid warming to slow cooling of the concrete inside the mass concrete. The influence of cement fineness on volume deformation of mass concrete under actual project conditions was obtained. These data can provide reference for construction and subsequent maintenance of actual engineering.

## 2. Materials and Methods

**Figure 2:**
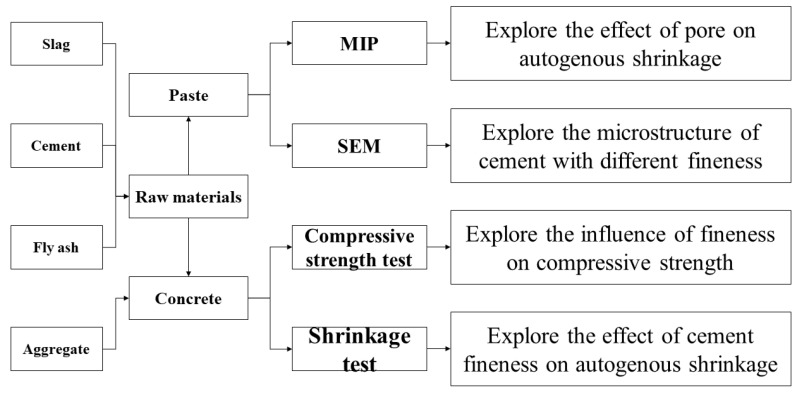
The flowchart diagram process followed in this study.

In this study, the compressive strength and autogenous shrinkage of concrete with different fineness were tested under the condition of variable temperature. The pore size and hydration degree were associated with the results of the above two tests through the paste microstructure and MIP results. The detailed flow chart is shown in Figure 2.

### 2.1. Raw Material and Mixing Ratio

#### 2.1.1. Raw Materials

**Figure 3:**
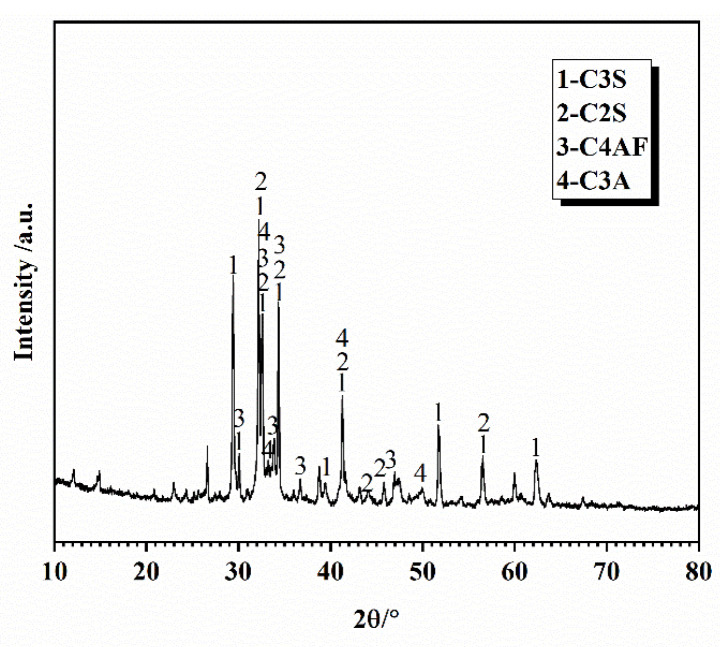
XRD pattern of cement.

**Figure 4:**
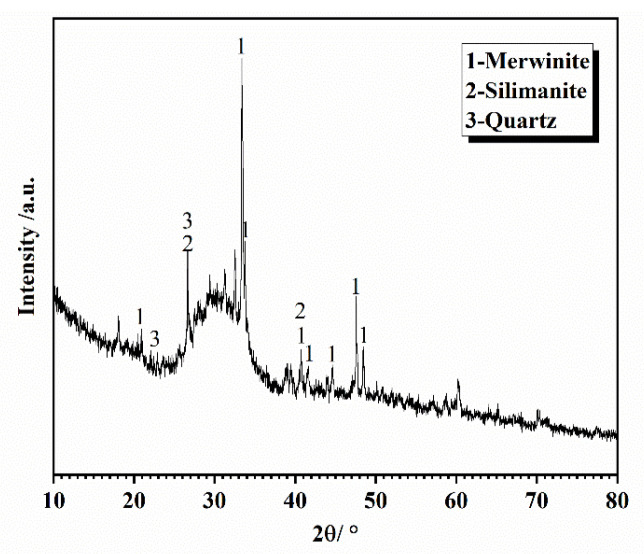
XRD pattern of slag.

**Figure 5:**
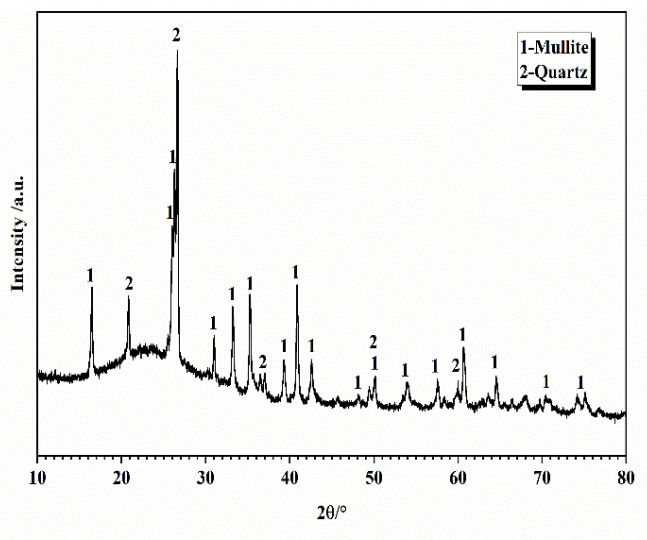
XRD pattern of fly ash.

**Figure 6:**
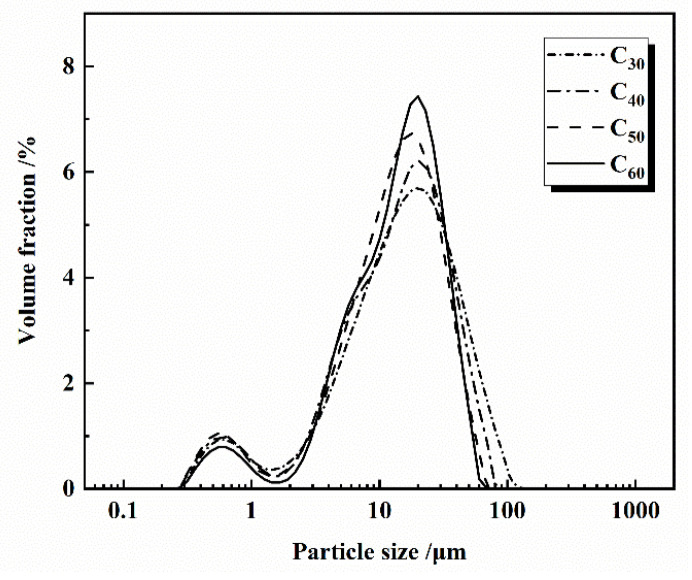
Particle size of cement.

The experimental P-grade silicate cement was homemade, using Jiyuan clinker (Henan, China) and gypsum from Nanjing Zhonglian (Nanjing, China). The four types of cement produced by ball milling for 30, 40, 50, and 60 min, respectively, are C_30_, C_40_, C_50_, and C_60_. The specific surface areas were measured by using Burr’s specific surface area tester (Jianyi Instrument Co., Ltd. Wuxi, China) as 300, 327, 373, and 377 m^2^/kg, and the fineness of the four types of cement was measured by using a negative pressure sieve analyzer as 12.21 μm, 7.62 μm, 4.18 μm, and 3.44 μm, respectively. The manufacturer is yarn screening factory (Zhejiang, China). The fineness of the four types of cement was 12.21 μm, 7.62 μm, 4.18 μm, and 3.44 μm, and the four cement numbers were C_30_, C_40_, C_50_, and C_60_. The experimental S95 mineral powder and secondary fly ash were sourced from Nanjing Pudi (Nanjing, China). The experimental high-performance polycarboxylic acid water reducing agent was sourced from Nanjing Sobute (Nanjing, China). The chemical composition of cement, mineral powder, and fly ash is shown in Table 1; the mineral composition of cement is shown in Figure 3; the slag is shown in Figure 4; the fly ash is shown in Figure 5; the particle size distribution of cement is shown in Figure 6.

#### 2.1.2. Mixing Ratio

The water–cement ratio directly determines whether or not self-desiccation shrinkage will occur, with H.E. Davis stating that for concrete with a water–cement ratio of 0.6–0.94, the size of self-desiccation shrinkage is between 1/5 and 1/10 of the total long-term shrinkage of these concretes [36]. Tazawa’s study showed that self-desiccation shrinkage only occurs when the water–cement ratio is less than 0.42 and that the autogenous shrinkage of concrete with a water–cement ratio of 0.3 has similar values to self-desiccation shrinkage [37,38]. Therefore, the water–cement ratio of 0.32 was chosen for the experimentally placed concrete, which is consistent with the water–cement ratio in the actual project. The fine aggregate fineness modulus was 2.7, and the concrete mix ratios are shown in Table 2.

### 2.2. Experimental and Test Methods

#### 2.2.1. Simulation of the Temperature Variation of Silicate Concrete

**Figure 7:**
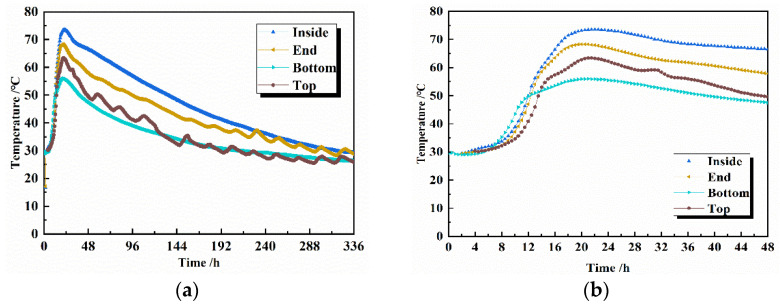
Concrete temperature change process of 336 h (**a**) and 48 h (**b**) in the actual project.

**Figure 8:**
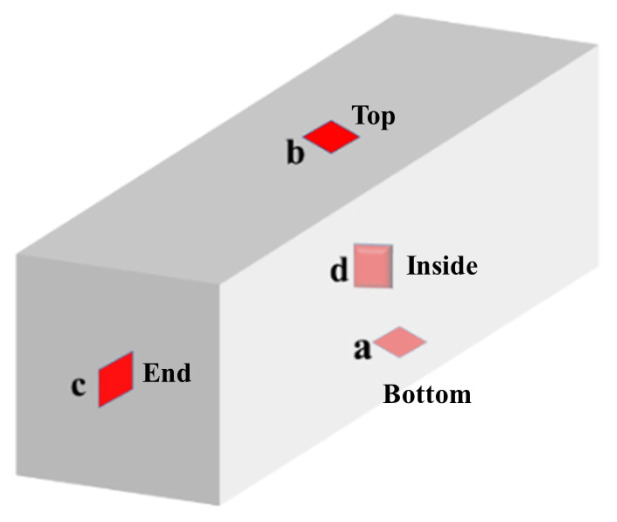
Temperature sampling points in the actual project.

**Figure 9:**
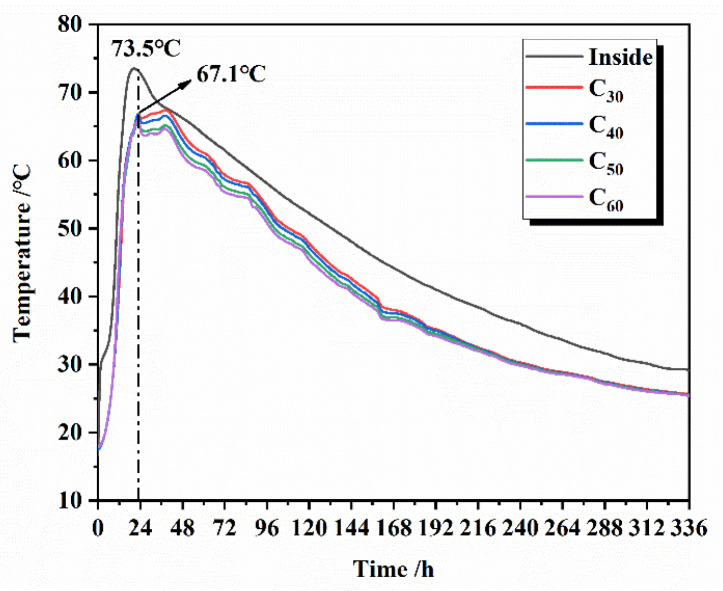
The simulated temperature change process for the experiment.

The temperature rise profile inside the concrete is simulated for actual situations, and the concrete specimens are maintained in a variable temperature curing chamber. The heating rate of the curing chamber is controlled in real time by the internal temperature of the specimen fed back from the strain gauge to match the temperature profile of the specimen as closely as possible to the actual project. The temperature data curve in the actual project is shown in Figure 7. The temperature peaks at the bottom (a), top (b), and end (c) are 55.9 °C, 63.4 °C, and 68.1 °C, respectively, all of which are lower than the 73.5 °C temperature peak inside (d) the middle of the concrete. The sampling points of concrete temperature in the actual project are shown in Figure 8. However, the time taken to reach the maximum temperature at all four locations is close, at around 18–20 h, and all cooled down from the maximum temperature to an ambient temperature in approximately 14 days. In order to reduce the influence of environmental factors, we chose the internal temperature profile to represent the temperature change history inside the concrete. The process of temperature change shown by the strain gauges placed in the concrete specimens during the experiment is shown in Figure 9, with a warming time of 18 h and a cooling time of 14 days. Since the time of the actual project is in summer and the simulated experiment is in autumn, the difference in season causes the overall temperature of the actual project to be higher than the overall temperature of the simulated experiment by about 6 °C. The heating and cooling rates of the simulated experiments were consistent with the temperature change history of the concrete in the middle of the actual project. The maintenance continued using PVC cling film to wrap the concrete specimen to ensure no moisture exchange with the outside world. The specimens were removed and tested for compressive strength after 3, 7, 28, and 60 days of maintenance.

#### 2.2.2. Compressive Strength Test of Silicate Concrete

**Figure 10:**
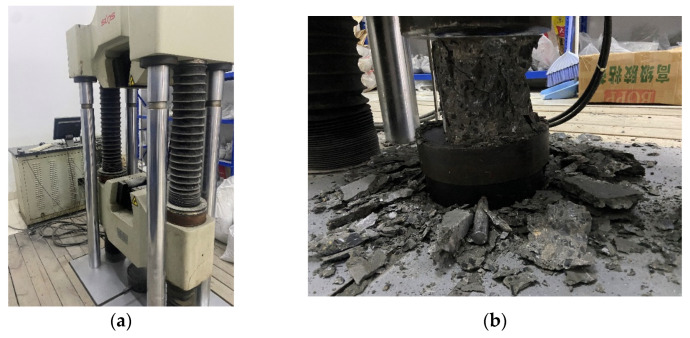
The concrete strength tester (**a**) and compressive strength specimen of concrete (**b**).

Compressive strength is one of the essential properties of concrete, and the value of early compressive strength is used to evaluate the degree of hydration of cement particles in concrete. The test method followed the Standard for Mechanical Test Methods of General Concrete GB/T50081-2002, using cubic compressive strength as the strength characteristic value of concrete. The concrete was sealed with PVC film as soon as it was poured into the mold to ensure it was in a simulated moisture-proof condition. We put the concrete into the curing box to simulate the internal temperature change of the concrete for 24 h and then demoulded it. After demoulding, we continued to put it into the curing box at variable temperatures. The concrete compressive strength tester (a) and the compressive strength specimen (b) are shown in Figure 10.

#### 2.2.3. Shrinkage Test of Silicate Concrete

**Figure 11:**
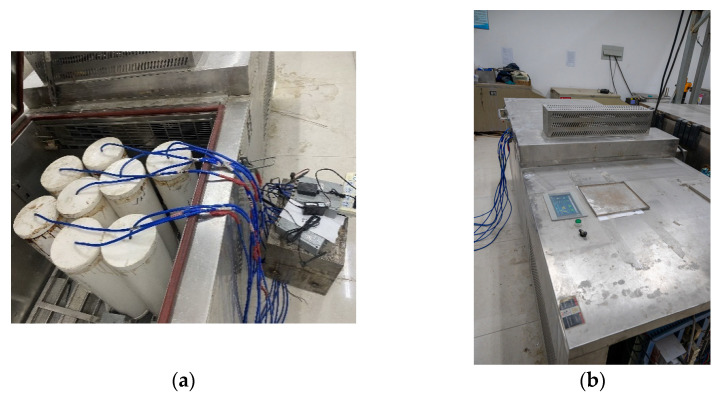
The device for measuring autogenous shrinkage (**a**) and variable temperature curing box (**b**).

The testing device for concrete autogenous shrinkage was composed of a PVC hollow pipe with a height of 500 mm and a radius of 160 mm with a VWS-15 vibrating string strainer made by Ge Nan Industrial Company (Nanjing, China) and a GDA16 full-function data acquisition instrument. The effective length of the specimen poured into the tube is 400 mm. A sealing cap with an inner diameter of 160 mm is coated with special PVC glue to seal the hollow tube’s end as the mold’s bottom. Mix four kinds of cement numbered C_30_, C_40_, C_50_, and C_60_ in batches, and then pour the concrete into the shrinkage test device, with the device numbers C_30_, C_40_, C_50_, and C_60_ in sequence. The concrete was poured to a height of 400 mm. During that time the strain gage was placed in the middle of the concrete to ensure that the gage takes uniform stress data, and 20 mm of liquid paraffin was poured into the upper part of the concrete after the concrete had set to insulate the upper part. This was covered with the same sealing lid as the bottom and the mold was used for measuring the autogenous shrinkage into the variable temperature maintenance box. The initial setting time of concrete was used as the starting point for measuring the autogenous shrinkage strain. The data collection device (a) and curing box (b) are shown in Figure 11.

#### 2.2.4. Pore Structure Test of Silicate Cement Paste

Mercury piezometer is one of the main instruments for measuring the pore structure of cement paste. Before pore structure testing, samples were dried under a vacuum at 105 °C for 4 to 5 h to remove capillary water and alcohol from the specimens. Under certain pressure, the mercury was pressed into the open hole in the cement paste. The stress and the volume of mercury pushed into the hole were continuously recorded during the test, ranging from 300 μm to 7 nm. The orifice diameter corresponding to each pressure can be calculated from Washburn’s [39] equation:

d = (−4γ × cosθ)/P(5)

P—the pressure required to press the mercury into the hole;

d—the pore diameter of the hole the mercury is pressed into;

γ—the surface energy of the cement;

θ—contact angle of mercury with the wall of the hole.

The strength of the cement paste and the pore structure are closely related. PoreMaster GT-60 automatic mercury compression meter was used to test the pore structure of four types of net cement pastes, the equipment is made by Quantachrome company (Boynton Beach, FL, USA). Cement paste pore size (d) is divided into four classes: less than 20 nm for harmless pores; 20~50 nm for less harmful pores; 50~200 nm for harmful pores; more than 200 nm for more harmful pores. The actual control of hardened cement slurry strength, permeability, and volume changes is the pore size distribution rather than the porosity. The water–cement ratio and the degree of hydration mainly govern the pore gradation. Pores larger than 50 nm mainly harm the strength and permeability of the cement paste; pores smaller than 50 nm primarily affect the drying shrinkage and creep of the cement paste.

## 3. Results and Analysis

### 3.1. Effect of Silicate Cement of Different Fineness on the Compressive Strength of Concrete under Different Temperature Conditions

**Figure 12:**
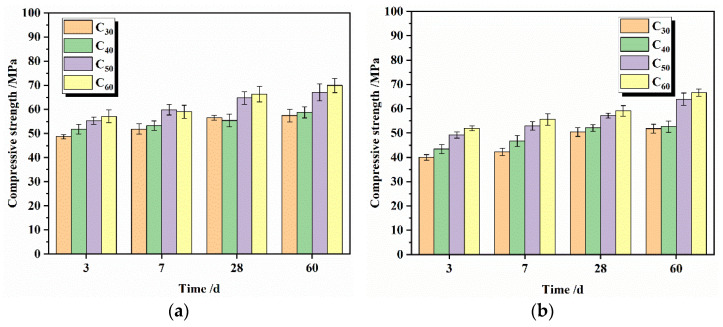
Compressive strength of concrete at different ages under changing temperature curing conditions (**a**) and 20 °C curing conditions (**b**).

The effects of silicate cement with different fineness on the compressive strength of silicate concrete are shown in Figure 12.

As can be seen from Figure 12, the compressive strength of concrete with different fineness increased due to the change in concrete curing temperature, especially the early strength. At 3 days, the compressive strength under variable temperature curing was, on average, 7.5 MPa higher than that under normal temperature curing, 5.5 MPa higher at 7 days, and only 4.75 MPa higher at 60 days. The effect of maintenance temperature on strength gradually decreases, and the variable temperature conditions are conducive to the rapid growth of concrete strength at early ages.

The compressive strength of concrete under a variable temperature curing environment is shown in Figure 12a. With the increase of silicate cement fineness, the early compressive strength of concrete gradually increases. The smaller the fineness of cement, the faster the early growth of compressive strength of concrete. The particles of 3 μm–30 μm are the main factors affecting the strength at each age. The proportion of particles of 3 μm–30 μm to the total particles in C_30_, C_40_, C_50_, and C_60_ is 68.5%, 73.47%, 81.17%, and 78.21%, respectively. At 3 days, the compressive strength of C_60_, the finest particle, reached 57.4 MPa, compared with 56.5 MPa for C_50_, 51.8 MPa for C_40_, and 48.7 MPa for C_30_. At 60 days, the compressive strengths reached 69.9 MPa, 66.3 MPa, 59 MPa, and 57.1 MPa, respectively. The compressive strength is negatively correlated with the fineness of cement particles, and the different concrete strength values at intermediate ages follow the above rule. 

The 3-day compressive strength of concrete for C_60_ is 85.29% of the 60-day compressive strength of concrete, the 3-day compressive strength of concrete for C_50_ is 87.80% of the 60-day compressive strength of concrete, the 3-day compressive strength of concrete for C_40_ is 85.22% of the 60-day compressive strength of concrete, and the 3-day compressive strength of concrete for C_30_ is 82.12% of the 60-day compressive strength of concrete. The finer the particle size of the cement, the higher the proportion of the 3-day compressive strength to the 60-day strength. As the age increases, the value of strength increases per unit of time decreases. The average compressive strength increase from 3 to 7 days was 2.75 MPa, with an average increase of 0.69 MPa per day. The average compressive strength increase from 7 to 28 days was 4.225 MPa, with an average increase of 0.22 MPa per day. The average compressive strength increase from 28 to 60 days was 3.1 MPa, with an average increase of 0.097 MPa per day.

There are two possible reasons for the above results. On the one hand, the finer the particles of silicate cement, the higher the hydration activity, and the easier it is to hydrate quickly in the early stage, generating more hydration products dense the structure of the concrete, and the strength grows fast [40]. With the gradual extension of the age, the concrete internal particles of coarse cement began to hydrate and continued to generate new hydration products but will not increase the strength of concrete as quickly as 3 days ago. On the other hand, the temperature change course of concrete is always above 60 °C during the 3-day age, which is similar to the conditions of high-temperature curing. The high temperature will promote the hydration of cement in concrete, rapidly increasing the strength of concrete within 3 days of age and gradually decreasing the temperature as the age increases. At 7 days, the temperature has already reached 40 °C, the promotion effect on cement hydration is limited, and the strength growth becomes slow. After 14 days, the temperature drops to room temperature, the temperature does not promote the hydration of the cement, and the strength growth course is the same as at room temperature.

### 3.2. Effect of Silicate Cement with Different Fineness on the Autogenous Shrinkage of Silicate Concrete

#### 3.2.1. Effect of Silicate Cement of Different Fineness on the Total deformation of Silicate Concrete

**Figure 13:**
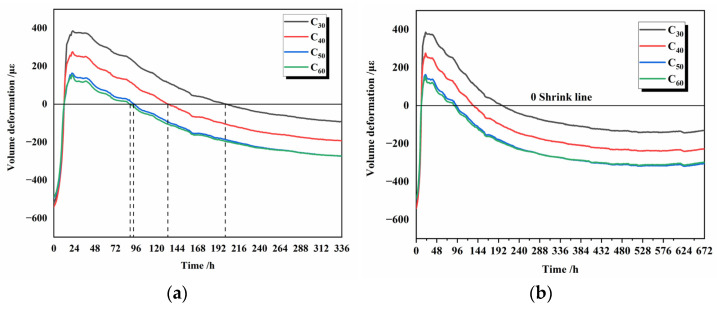
Volume deformation of cement with different fineness at 14 d (**a**) and 28 d (**b**).

The effect of different fineness of silicate cement on the total volume deformation of silicate concrete is shown in Figure 13. The test specimens were sealed during the measurement age, and the reference zero point for measuring the concrete strain was 12 h. A set of stress data were collected every one h using the collection equipment until 28 days. The collected stress data were converted into a strain, indicating the total volume deformation data of the concrete according to the formula.

ε_t_ = k × (F_t_ − F_0_) + b × (T_t_ − T_0_) (6)

εt—the total volume deformation of silicate concrete;

k—the measurement sensitivity of the strain gauge in 10^−6^/F;

F_t_—the measured value of the strain gauge strain at moment t in F;

F_0_—the reference value of the strain gauge for concrete deformation in F;

T_t_—the measured value of the temperature of the strain gauge at the moment t, in °C;

F_0_—the temperature reference value of the concrete deformation in °C;

b—the coefficient of thermal expansion of the strain gauge, provided by the manufacturer, 13 × 10^−6^/°C for this experiment.

Figure 13 shows that the strain data for all four types of concrete undergo a process of increasing and then decreasing until negative values, indicating that the macroscopic total volume change of concrete is expansion followed by contraction. Two main factors cause early-age swelling. One is the thermal expansion due to the increase in concrete temperature, and the total strain curve in Figure 13 possesses the same trend as the temperature curve of concrete. The other is due to the reaction of substances such as f-CaO and MgO contained within the raw material with water, which causes volume expansion. Both of them together lead to an increase in the total strain data. The strains of the four concrete reached their respective highest values near 12 h after the zero point of autogenous shrinkage, i.e., 24 h after mixing, as 385 με, 375 με, 162 με, and 154 με, respectively, with the gradually increasing temperature playing a dominant role in this expansion process.

After the peak temperature, as the temperature decreases, the degree of influence of temperature on the total strain gradually decreases, and the effect of the contraction of the cement itself on the overall volume deformation gradually increases. The total strain data for the four concrete became negative at 200 h, 133 h, 93 h, and 89 h, respectively. Eventually, the different cement fineness caused different times of starting shrinkage and total shrinkage of the concrete. The total shrinkage strain for different fineness of cement under variable temperature conditions was from small to large for C_30_, C_40_, C_50_, and C_60_, corresponding to −92 με, −193 με, −273 με, and −274 με at 14 days, respectively, the line 0 in Figure 13 is the reference line of total volume deformation, and the shrinkage of concrete gradually stabilized after more than 14 days. The finer the cement particles, the earlier the corresponding concrete begins to shrink in volume under the same variable temperature conditions, and the greater the total shrinkage at the same age.

#### 3.2.2. Effect of Different Fineness of Silicate Cement on the Autogenous Shrinkage of Silicate Concrete

**Figure 14:**
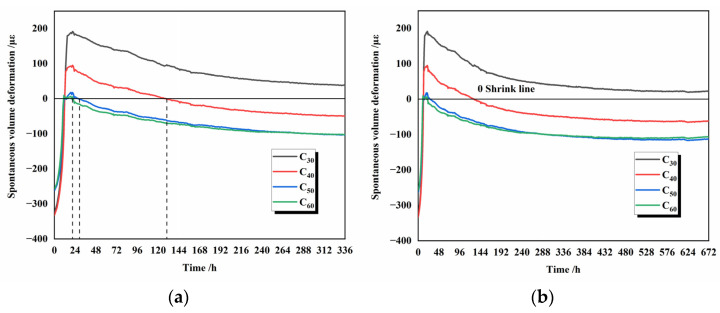
Spontaneous volume deformation of cement with different fineness at 14 d (**a**) and 28 d (**b**).

The effect of different fineness of silicate cement on the total volume deformation of silicate concrete is shown in Figure 14. The stress is first converted into strain data by using the calculation formula. Following that, the concrete’s autogenous shrinkage strain data were obtained by deducting the thermal expansion of the concrete due to temperature.

ε_t_ = k × (F_t_ − F_0_) + (b − α) × (T_t_ − T_0_) (7)

ε_t_—the autogenous shrinkage deformation of silicate concrete;

k—measurement sensitivity of the strain gauge in 10^−6^/F;

F_t_—the measured value of the strain gauge at the moment t, in F;

F_0_—the reference value of the strain gauge for concrete deformation in F;

T_t_—the measured value of the temperature of the strain gauge at the moment t, in °C;

F_0_—the temperature reference value of the concrete deformation in °C;

b—the coefficient of thermal expansion of the strain gauge, provided by the manufacturer, 13 × 10^−6^/°C for this experiment;

α—the coefficient of thermal expansion of silicate concrete, the concrete is mainly composed of basalt and natural sand, α is taken as 7 × 10^−6^/°C.

The greater the fineness of the silicate cement at 28 days of age, the earlier the concrete starts to self-shrink. Because the finer the particles in the early stage, the more contact area between cement and water, the faster hydration reaction rate can be obtained, and the greater the number of hydration products C-S-H gel generated per unit of time. At 7 days, the finest-grained group of concrete C_60_ microstrain was −80 με. The microstrains of concrete in groups C_30_, C_40_, and C_50_ are 72 με, −19 με, and −74 με, respectively, the line 0 in Figure 14 is the reference line of autogenous shrinkage, which differ from group C_60_ by 152 με, 61 με, and 6 με, respectively. It can be seen from Figure 14 that the concrete autogenous shrinkage growth starts to slow down after 7 days, and this trend is 7 days ahead of the total volume deformation. At this time, the autogenous shrinkage microstrain of the two groups of concrete C_60_ and C_50_ with the finest particles are close. This trend is maintained during the age period of 7 days–28 days. This indicates that there are other factors besides the fineness of the cement that affect the size of the autogenous shrinkage of the concrete after 7 days of age. After 14 days, the autogenous shrinking microstrain of the concrete in the three groups was unchanged, and the hydration reaction was finished.

Figure 13 and Figure 14 show that temperature and autogenous shrinkage affect the macroscopic volume deformation of concrete at different stages throughout the hydration process, respectively. At 0–1 day, the gradual rise in temperature has the same trend as the macroscopic volume deformation, and the high temperature above 40 °C within this phase will also accelerate the hydration reaction of cement. At this time, the internal temperature is the main factor affecting the macroscopic volume deformation of concrete. At 1–7 days, Figure 8 shows that the slope of the total strain decrease becomes slower at 7 days because the hydration reaction is finished at this time, and the strain value of autogenous shrinkage is unchanged, which does not affect the overall macroscopic deformation. At 7–14 days, the internal temperature of concrete drops from 45 °C. At 14 days, it drops to room temperature, the effect of temperature on macroscopic volume deformation gradually decreases, and the overall volume deformation remains the same.

### 3.3. Effect of Different Fineness of Silicate Cement on the Pore Size of Cement Paste

**Figure 15:**
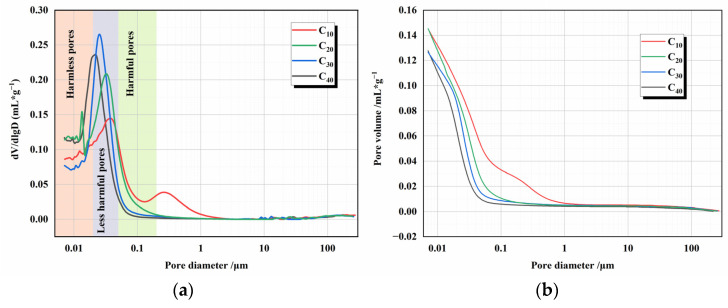
MIP results for different fineness cement under changing temperature conditions (**a**,**b**).

The pore structure of cement slurries with different fineness was determined by mercury intrusion porosimetry (MIP). The results of the tests are shown in Figure 15. The porosity and pore size distribution of cement slurry at 3 days are shown in Table 3. It can be seen that there is not much difference in the porosity of the cement paste of different fineness due to the effect of variable temperature. The higher temperature inside the concrete during warming can promote the formation of the concrete structural skeleton.

However, the smaller the value of cement fineness, the more significant the proportion of total pores occupied by harmless pores. The compressive strength of concrete of this fineness is positively correlated with the proportion of harmless pores to total pores. The larger the proportion of harmless pores, the smaller the proportion of multi-harmful pores. The higher the corresponding compressive strength of the concrete at the same age. This is because in the early hydration process, the finer the cement, the smaller and denser the hydration products can be generated per unit of time. These hydration products can fill the cavities inside the concrete faster, enhance the denseness of the concrete, reduce the overall size of the holes, and effectively reduce the percentage of multi-harmful holes. The skeleton and filler work together to improve the compressive strength of the concrete.

## 4. Microscopic Analysis of Cement Paste

**Figure 16:**
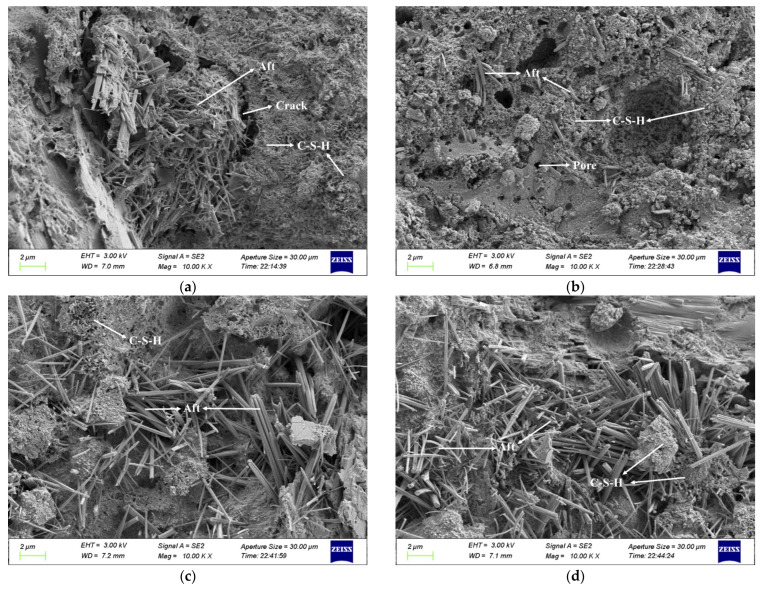
Microstructure of cement paste numbered C_30_ (**a**), C_40_ (**b**), C_50_ (**c**), and C_60_ (**d**).

The study characterized the internal structure of cement paste with different fineness using SEM analysis. The SEM images of cement paste numbered C_30_, C_40_, C_50_, and C_60_ in a, b, c, and d of Figure 16, respectively.

The comparison of SEM images showed that as the fineness of cement particles gradually increased, the more completely the cement particles hydrated, the more flocculent hydration products were formed, and the overall texture became dense. In addition, the internal structure of the cement paste was improved in terms of pore size, microcrack formation, and filling. On the other hand, it can be observed in Figure 16 that with the increase of cement fineness, the distribution of needle-like calcium alumina in the microstructure cement paste is gradually dispersed. The number is increasing, with needle-like calcium alumina as the framework and hydrated calcium silicate as the binder. This structure and hydrated calcium silicate can lead to a higher compressive strength of concrete.

Autogenous shrinkage in cement paste is determined by chemical shrinkage, self-desiccation shrinkage with pores, and cracks after hardening. From Figure 15 we can see that as the cement particles become finer, the cement can hydrate more fully and the generated C-S-H can fill the original pores or cracks better, reducing the pore volume in the cement paste. At the same time, smaller pore sizes produce greater self-desiccation shrinkage, and they both make the shrinkage more pronounced.

## 5. Conclusions

The most significant conclusions of this study are summarized as follows:

(1) Under variable temperature conditions, the finer the cement particles, the faster the compressive strength of concrete grows. When the test age increases, the less the compressive strength of concrete increases per unit of time.
(2) The finer the cement particles are, the greater the total volume deformation and self-shrinkage of the concrete, and the earlier the shrinkage begins during the 28-day temperature change course.
(3) Throughout the variable temperature phase, temperature is not the only dominant factor affecting the total volume deformation of concrete. In 1–7 days, the self-shrinkage produced by cement hydration becomes the main factor affecting the total volume, so the study of the total volume deformation of concrete under variable temperature conditions should be divided into three stages.
(4) The high-temperature environment at an early age promotes the hydration reaction of cement and reduces the difference of porosity of net cement paste with different fineness, but the smaller the fineness of cement, the greater the proportion of harmless pores in cement paste and the smaller the proportion of multi-harmful pores. The compressive strength of concrete at the same age is mild with this phenomenon.

## Figures and Tables

**Table 1:** Chemical analysis of raw materials (wt%).

Materials	SiO_2_	Al_2_O_3_	Fe_2_O_3_	TiO_2_	CaO	MgO	SO_3_	K_2_O	Na_2_O	LOI	Total
Cement	18.55	3.95	3.41	0.207	65.32	1.01	2.78	0.721	0.176	2.88	99.004
Fly ash	44.06	42.06	2.91	1.64	3.80	0.395	0.754	0.487	0.155	2.48	98.741
Slag	33.39	11.89	0.630	0.483	41.51	8.82	1.32	0.527	0.672	−0.28	98.962

**Table 2:** Concrete mix ratios (kg/m^3^).

Cement	Mineral Powder	Fly Ash	Water	Fine Aggregate		Coarse Aggregate			Water Reducer
				0–2.36	2.36–5	5–10	10–20	20–30	
360	40	90	157	136	544	220	550	330	9.8

**Table 3:** Porosity and pore size distribution of different cement paste at 3 d (wt%).

Specimen	Porosity	Percentage of Each Type of Pore in the Total Pore			
		Harmless Pores	Less Harmful Pores	Harmful Pores	Multihazardous Pores
		0–0.02	0.02–0.05	0.05–0.2	>0.2
C_30_	24.51	29.16	35.82	18.1	16.92
C_40_	21.47	31.96	56.89	5.71	5.44
C_50_	24.2	36.4	46.87	12.07	4.66
C_60_	21.66	57.71	35.14	3.32	3.83

## Data Availability

The data presented in this study are available on request from the corresponding author.
